# Melatonin Synergizes With Methylprednisolone to Ameliorate Acute Spinal Cord Injury

**DOI:** 10.3389/fphar.2021.723913

**Published:** 2022-01-13

**Authors:** Jiaqi Bi, Peiyu Sun, Erwei Feng, Jianxiong Shen, Chong Chen, Haining Tan, Zheng Li, Youxi Lin

**Affiliations:** ^1^ Department of Orthopedic Surgery, Peking Union Medical College Hospital, Chinese Academy of Medical Sciences and Peking Union Medical College, Beijing, China; ^2^ Emergency Department, SongBei Hospital of the Fourth Hospital Affiliated with Harbin Medical University, Harbin, China; ^3^ Postdoctoral Workstation, Harbin Children’s Hospital, Harbin, China; ^4^ Department of Orthopedics, Bejing Hospital of Traditional Chinese Medicine, Beijing, China; ^5^ Department of Spine Surgery, Orthopedics Center of Guangdong Provincial People’s Hospital and Guangdong Academy of Medical Sciences, Guangzhou, China; ^6^ Department of Orthopedic Surgery, Beijing Friendship Hospital, Capital Medical University, Beijing, China; ^7^ Department of Orthopedic Surgery, Beijing Tiantan Hospital, Capital Medical University, Beijing, China

**Keywords:** melatonin, methylprednisolone, spinal cord injury, factorial design, PI3K-AKT1 pathway

## Abstract

Methylprednisolone (MP) is the drug of choice for treating spinal cord injury (SCI), but the aggressive dosage regimen used often results in adverse side effects. Therefore, MP should be combined with other drugs to lower the required dose. Melatonin is effective in alleviating nerve damage and inhibiting axonal degeneration. The combination of melatonin and half-dose methylprednisolone (HMP) for spinal cord injury treatment has never been reported. In this study, we established a rat model of T9 spinal cord injury by the Allen’s method and assessed the synergistic therapeutic effects of melatonin and HMP by factorial design. Our results demonstrated that melatonin could synergize with HMP to ameliorate acute SCI through PI3K-AKT1 pathway. Combining melatonin with HMP significantly reduced the standard-dose of methylprednisolone and limited its adverse reactions, representing a promising option for treating acute SCI.

## Introduction

The spinal cord is one of the most complex and specialized tissues in the human body and is very sensitive to ischemia, hypoxia, and trauma (Karsy and Hawryluk, 2019). This is pathologically characterized by nuclear condensation, loss of Nissl bodies, and axonal degeneration, amongst others (Simon et al., 2016, Bothwell, 2017). Simultaneously, neuronal necrosis can result in the release of intracellular content, which can induce secondary spinal injury (Park et al., 2019). Secondary injury is the leading cause of subsequent glial cell filling and functional impairment (Durna et al., 2019). Therefore, approaches to alleviate spinal cord injury (SCI) include preventing apoptosis, inhibiting Nissl body lysis, maintaining cell homeostasis, and promoting axon growth.

Methylprednisolone (MP) is currently the drug of choice for the treatment of SCI in clinical practice and is recommended by the National Acute Spinal Cord Injury Study (NASCIS) (Bracken et al., 1997). MP has an effective therapeutic window, in which it can reduce the toxicity and neuroinflammation within the spinal cord (Vidal et al., 2018, Spinal Cord Injury (I), 2016). However, an aggressive dosage regimen is required to maintain the MP concentrations within this therapeutic window. This high dosage can lead to several complications, such as systemic fungal infections and allergies, representing a challenge in the widespread use of MP for SCI(Falavigna et al., 2018, Miękisiak et al., 2019). Moreover, clinical reports show that these contraindications are easily masked by other symptoms and may, therefore, cause serious harm to the patient (Jain et al., 2015). Recent studies suggest that treating acute spinal cord injury with MP primarily protects axons, while its protective effect towards cellular organelles is weak.

Melatonin (N-acetyl-5-methoxytryptamine, MT) has various neuroprotective effects and can be enriched in the cerebrospinal fluid (Morton et al., 2020, Corpas et al., 2018). MT has also been shown to be effective in the treatment of SCI, with beneficial effects on neuron cell bodies and synapses (Areti et al., 2017, Park et al., 2012). MT is fat-soluble and can easily pass through cell membranes, thereby protecting mitochondria and other organelles; however, MT can also cause several adverse reactions when the dose is high (Mendivil-Perez et al., 2017; Pérez-González et al., 2019). Many studies have shown that the protective effects of MT on the spinal cord are due to the scavenging of oxygen free radicals, protecting against ischemia and hypoxia of nerve tissue, promoting regeneration of the peripheral nerve myelin sheath, and stimulating various anti-apoptotic signaling pathways (Shen et al., 2017; Zhang et al., 2018; Xu et al., 2019). The combination of MT and MP at standard dose displays synergistic inhibitory effects towards lipid peroxidation after SCI(Kaptanoglu et al., 2000, Cayli et al., 2004). Therefore, this study aimed to explore the synergistic effects of MT and HMP in the amelioration of SCI in a rat model to amend the optimal dose and reduce adverse drug events.

## Methods

### Animals

The 240 animals used in the experiment were 8–10 week old Sprague-Dawley (SD) rats weighing ∼250 g were purchased from the SPF Biotechnology Company (Beijing, China). Rats were housed under a 12 h light/dark cycle at 25°C ± 5°C and 60–70% humidity and had access to water and food ad libitum. All animal experiments were approved by the Ethics Committee of Peking Union Medical College Hospital.

### Establishment and Intervention of Spinal Cord Injury in Rats

The rat SCI model was established as previously reported (Caudle et al., 2015). Briefly, the rats were anesthetized by intraperitoneal injection of 50 mg/kg 1% sodium pentobarbital. A longitudinal midline incision was made with T9 at the center before the spinous process, and the lamina of T8-10 was exposed and rinsed with normal saline. A 10 g hammer was dropped freely from a height of 25 mm to hit the T9 spinal cord, and then the spinal cord was pressed for 1 min. A small amount of muscle and skin were excised, and the tissue was sutured. Animals that were completely paralyzed below the injury site were included in the experiment. The bladder was pressed once in the morning, midday, and evening until the bladder regained its urination reflex. The control (CTR) group was anesthetized, the T9 segment of the spinal cord was exposed after laminectomy, no other treatment, and then the skin was sutured. Rats did not receive any spinal damage.

Rats were randomly divided into 6 groups of 40 animals each: CTR group (as a negative control), SCI group (as an untreated control), MT group (Melatonin, 15 mg/kg, i.p), HMP group (Half-dose methylprednisolone, 15 mg/kg, i.p), COM group (Melatonin, 15 mg/kg, i.p and methylprednisolone, 15 mg/kg, i.p), and MP group (methylprednisolone, 30 mg/kg, i.p). Rats in the CTR and SCI groups were injected with 1 ml of physiological saline solution containing 1% ethanol. Melatonin (SIGMA-ALDRICH, St Louis, MO, United States), methylprednisolone (Pfizer Manufacturing Belgium NV). Movement and sensory recovery of all the animals were examined 1, 3, 7, and 14 days after model establishment. Six rats from each group were sacrificed on each test day, and the T8-T9 segments of the spinal cord were taken for subsequent experiments.

### Nissl Body Staining

Spinal cord T8-T9 segments were fixed in 4% formaldehyde for 4 h and embedded in paraffin. The sections were stained in 1% crystal violet for 10 min, differentiated in 95% alcohol, and Nissl bodies were observed (Normal optical microscope, NIKON, Japan). Three 40x visual fields were randomly selected and imaged. The neurons in each visual field were quantified, and an average was taken. Integrated intensities were quantitated using the Image Pro Plus 6.0 software (Media Cybernetics, Silver Spring, MD, United States).

### TdT-Mediated dUTP Nick-End Labeling Staining

TUNEL staining was performed following the manufacturer’s instructions (Tunel test kit, Roche, Swiss). The tissue sections were observed under an inverted microscope and imaged. Three 40x fields of view were randomly selected for each sample. The images were analyzed using Image-Pro Plus 6.0 software, and the spinal neurons were quantified. Positive cells displayed brown/yellow cytoplasm and blue nuclei. Apoptosis rate (%) = (number of positive cells/total cells) × 100.

### RNA Extraction and Real-Time Quantitative PCR (RT-qPCR) Analysis

Spinal cord tissue (1 cm in length) from the injured site was washed with cold PBS to remove blood, cut into small pieces, and homogenized with 1 ml Trizol reagent on ice for 10 min. The homogenized mixture was centrifuged at 12,000 rpm for 10 min before 250 μl of chloroform was added, and the mixture was centrifuged again. Next, 0.8 times volumes of isopropanol was added to the supernatant at −20°C for 15 min. After centrifugation, the precipitate was washed with 75% ethanol, and then DEPC water was used to dissolve the RNA. The reverse transcription and detection of RNA amplification were performed according to the manufacturer’s instructions (Servicebio technology CO.,LTD., Wuhan, China). The primers and reaction conditions are shown in Table 1.

**TABLE 1:** Specific primers used for real-time PCR analysis.

Primer	Sequence
GAP-43	5′-CCA​ACG​GAG​ACT​GCA​GAA​AGC-3′
	3′-GTC​AGC​CTC​GGG​GTC​TTC​TTT-5′
Synapsin I	5′-GAA​GTT​CTT​CGG​AAT​GGG​GTC-3′
	3′-GTC​AGC​CTC​GGG​GTC​TTC​TTT-5′
NF-200	5′-TGC​TCG​GTC​AGA​TTC​AGG​GC-3′
	3′-GAG​CGC​ATA​GCA​TCC​GTG​TT-5′
PSD-95	5′-GGT​TCC​ATC​GTT​CGC​CTC​TA-3′
	3′-GCA​ATG​CTG​AAG​CCA​AGT​CCT-5′
GAPDH	5′-CTG​GAG​AAA​CCT​GCC​AAG​TAT​G-3′
	3′-GGT​GGA​AGA​ATG​GGA​GTT​GCT-5′

### Western Blotting

Expression of GAP-43, synapsin I, NF-200, PSD-95, PI3K p85, PTEN, p-PDK1, PDK1, p-AKT1, AKT, p-NF-κB p65, and NF-κB p65 was evaluated by Western blot analysis. Spinal cord tissue homogenate was incubated in a lysate buffer containing protease inhibitors for 20 min on ice.

The collected supernatant was centrifuged at 14,000 rpm for 10 min, and the protein concentration was quantified using a BCA assay kit according to the manufacturer’s instructions (Servicebio technology CO., LTD., Wuhan, China). The supernatant was diluted to a uniform concentration of 10 mg/ml by adding a protein extraction reagent. The loading buffer (5X) was added to the sample (1:4) and boiled for 15 min.

Proteins were separated on a polyacrylamide gel 4–12% and transferred to a PVDF (polyvinylidene difluoride) membrane with wet transfer. The PVDF membranes were blocked using TBST containing 5% non-fat milk at room temperature for 2 h. The primary antibody diluted by 1:5,000 was added to the PVDF membrane and incubated at 4°C overnight. The membrane was washed three times with TBST before the secondary antibody diluted by 1:1,000 was added and incubated at 37°C for 2 h. Proteins in the PVDF membrane were quantified using enhanced chemiluminescence (CW Biotech) and analyzed using Image Quant TL software (GE, Sweden).

### Statistical Analysis

Data analysis was performed using SPSS26.0 software, and data were presented as mean ± standard deviation. Statistical significance was assessed using one-way ANOVA. LSD and S-N-K methods were used for multiple comparisons when variances were uniform, and Kruskal-Wallis H test with K independent sample test was used when variances were uneven. *p* < 0.05 was used as the criterion for statistical significance.

Multivariate factorial design statistical analysis was conducted using the SAS9.3 international standard statistical programming software. The data were described as mean ± standard deviation. The statistical analysis methodology used was three-factor factorial design variance analysis. When the interaction was statistically significant, the individual effects were analyzed. After analyzing the interaction and independent effects, *p* < 0.01 was used as the criterion for statistical significance.

## Results

### A Combination of MT and MP is Less Effective at Maintaining the Number of Nissl Bodies Than the Standard Dose of MP

**FIGURE 1:**
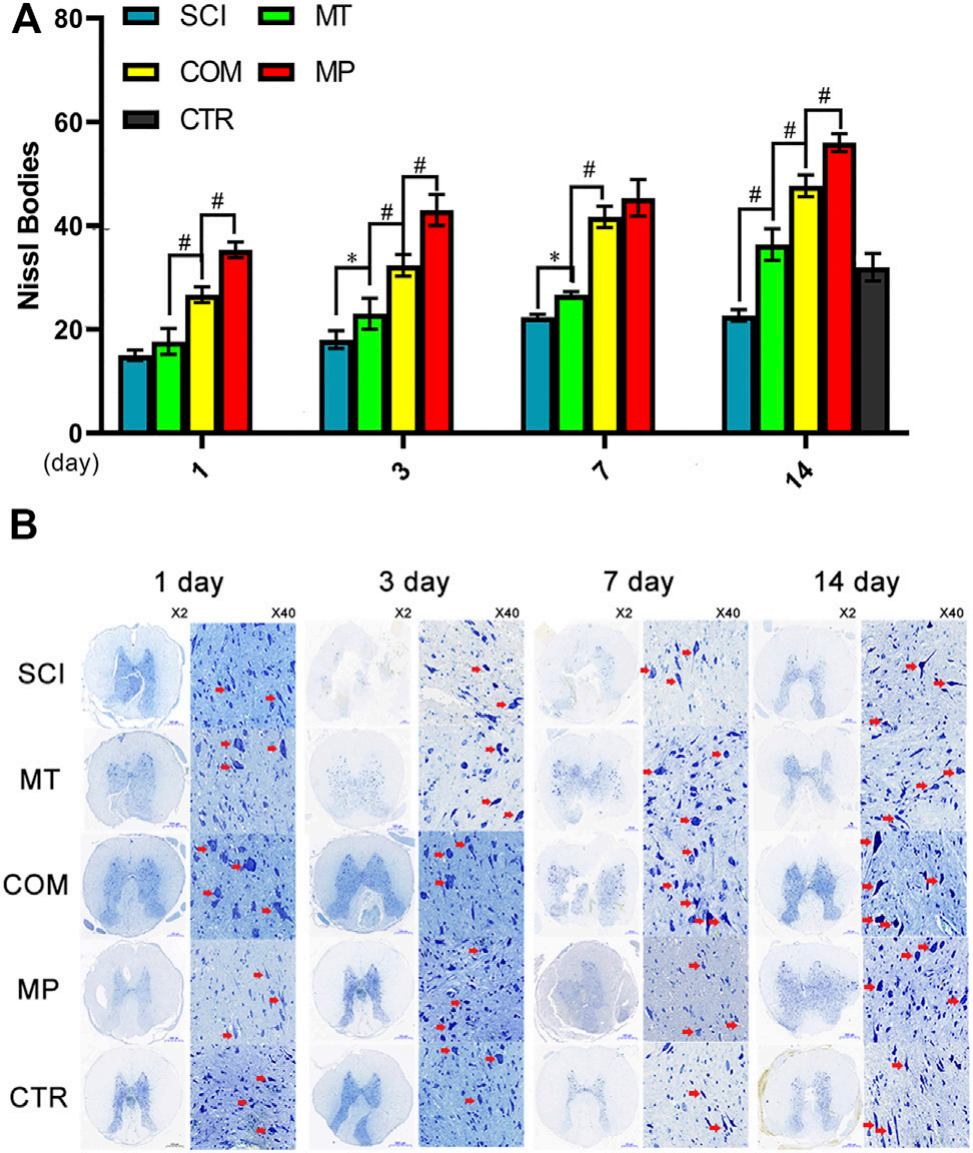
The Nissl body number in neuronal samples from the treatment groups. **(A)**: The number of Nissl bodies were quantified on the 1st, 3rd, 7th, and 14th day in neuronal samples taken from control rats (CTR, The average of the 4 sampling time points), rats with SCI (SCI), rats treated with melatonin (MT), methylprednisolone (MP), or a combination of MT with a low dose of MP (COM). **(B)**: Representative images of neuronal samples stained for the presence of Nissl bodies with crystal violet. The red arrow indicates the intact Nissl bodies. Error bars represent the standard deviation. *p < 0.05, #*p* < 0.01 (*n* = 6).

When the nerve cell body is injured, the first thing that appears is a decrease in Nissl bodies. The ruptured Nissl bodies release unassembled protein, which accelerates cell apoptosis and axon ulceration. Compared with the SCI group, the MT group showed a significant therapeutic effect on Nissl bodies (*p* < 0.05, *p* < 0.01). However, the therapeutic effect was not better compared to the CTR group until the 14th day. A combination of MT and half the standard dose of MP (COM group) maintained the number of Nissl bodies compared to MT alone (*p* < 0.01) from the 1st day after treatment (*p* < 0.01), and on the 3rd day, the number of Nissl bodies was higher compared to the CTR group. However, the number of Nissl bodies in the COM group was significantly lower than in rats in the MP group on days 1, 3, and 14 (*p* < 0.05; Figure 1A). At day 7, MT maintained Nissl bodies stable at day 7 and the COM group showed an adequate therapeutic effect. The therapeutic efficacy of the combination therapy in the COM group was similar to that observed in the MP group, but only on day 7 (Figure 1B).

### Apoptosis Inhibition by MT and MP Combined Was Comparable to the Standard Dose of MP Alone

**FIGURE 2:**
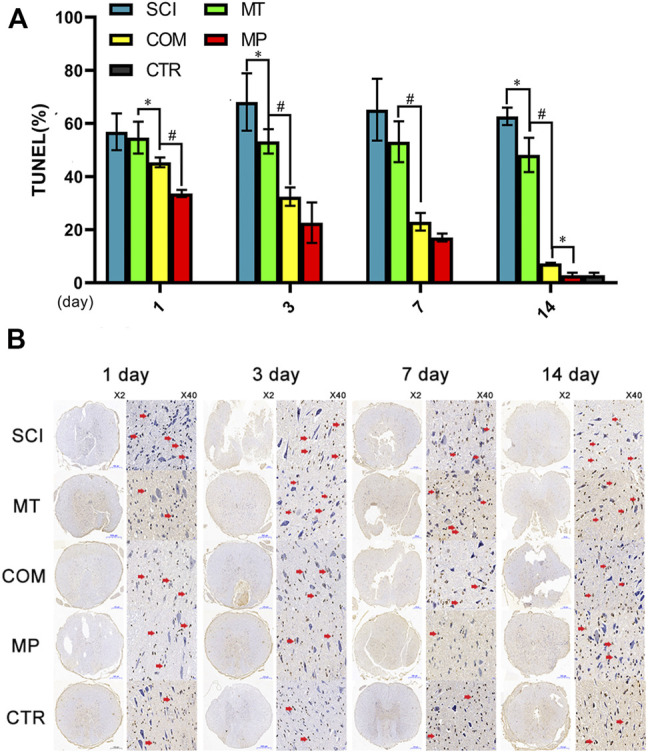
The number of apoptotic nerve cells among different groups. **(A)**: The number of apoptotic nerve cells were quantified on the 1st, 3rd, 7th, and 14th day in neuronal samples taken from control rats (CTR, The average of the 4 sampling time points), rats with SCI (SCI), rats treated with melatonin (MT), methylprednisolone (MP), or a combination of MT with a low dose of MP (COM). **(B)**: Representative images of TUNEL stained nerve cells. The red arrow in the TUNEL image indicates nuclear shrinkage. Error bars represent the standard deviation. *p < 0.05, #*p* < 0.01 (*n* = 6).

Increased apoptosis of nerve cell bodies significantly affects neuronal function. Compared with the negative control group, the MT group displayed significantly decreased apoptosis from day 3 to day 14 after treatment (*p* < 0.05; Figure 2A). By day 14 of treatment, the MP group exhibited an apoptosis level comparable to the control group (*p* > 0.05). However, rats in the COM group displayed similar apoptosis levels to the MP group on days 3 and 7 (Figure 2A). A similar result could be seen for the resistance to cell body shrinking and nuclear condensation, with the COM group displaying similar efficacy to the MP group (Figure 2B).

### The Combination of MT and MP Could Activate the PI3K-AKT1 Anti-Apoptotic Pathway Comparable to the Standard Dose of MP

**FIGURE 3:**
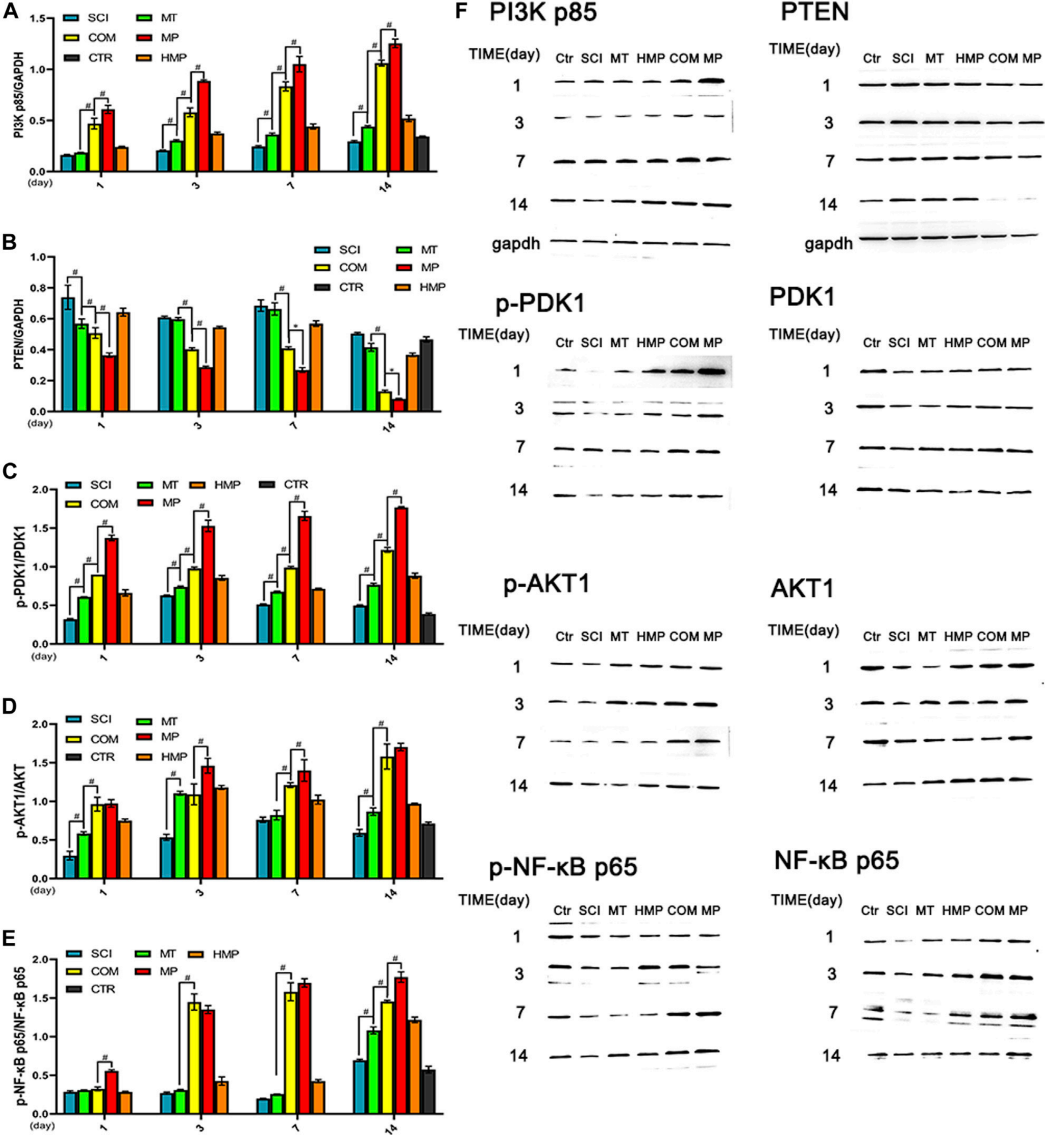
Western blot analysis of key proteins in the PI3K-AKT pathway in rats with SCI. **(A–E)**: The relative protein expressions of PI3K p85, PTEN, phospho-PDK1, phospho-AKT1, phospho-NF-κB p65 on the 1st, 3rd, 7th, and 14th days post-injury (*n* = 3); **(F)**: Representative blots of PI3KP85, PTEN, phospho-PDK1, phospho-AKT1, phospho-NF-κB p65. *p < 0.05, #*p* < 0.01. CTR group result is average of the 4 sampling time points.

The PI3K-AKT1 signaling pathway is highly related to the anti-apoptotic ability of cells. The PTEN, AKT1, and NF-κB proteins in the signaling pathway all play a vital role in neuronal apoptosis. Compared with the SCI group, we found that the MT group started displaying an upregulated expression of PI3K p85 protein from day 3. At day 7, the expression was higher than in the CTR group. The expression of PI3K p85 and PDK1 in rats from the COM group was lower than the MP group (*p* < 0.01; Figures 3A,C,F). The PTEN protein concentration of the MT group and the SCI group only differed on day 1 (*p* < 0.01; Figure 3B). The PTEN protein level in the COM group decreased significantly by the 3rd day, but it was still higher when compared with the MP group at all time points (Figures 3B,F). This suggested that stimulation of the anti-apoptotic pathway in rats from the COM group was more significant than following treatment with MT alone. Rats in the COM group showed similar Akt activation to rats in the MP group, and COM groups displayed higher Akt activation levels than rats in the MT group. MT treatment was shown to induce Akt pathway activation on the 3rd day. The MT group showed an increase in NF-κB protein expression on day 14, possibly due to the influence of the PTEN protein. The COM and MP groups had similar NF-κB protein expression on days 3 and 7 (Figures 3D–F).

### The Combination of MT and MP Reduced Axonal Degeneration by Regulating Axon-Related Nerve Proteins

**FIGURE 4:**
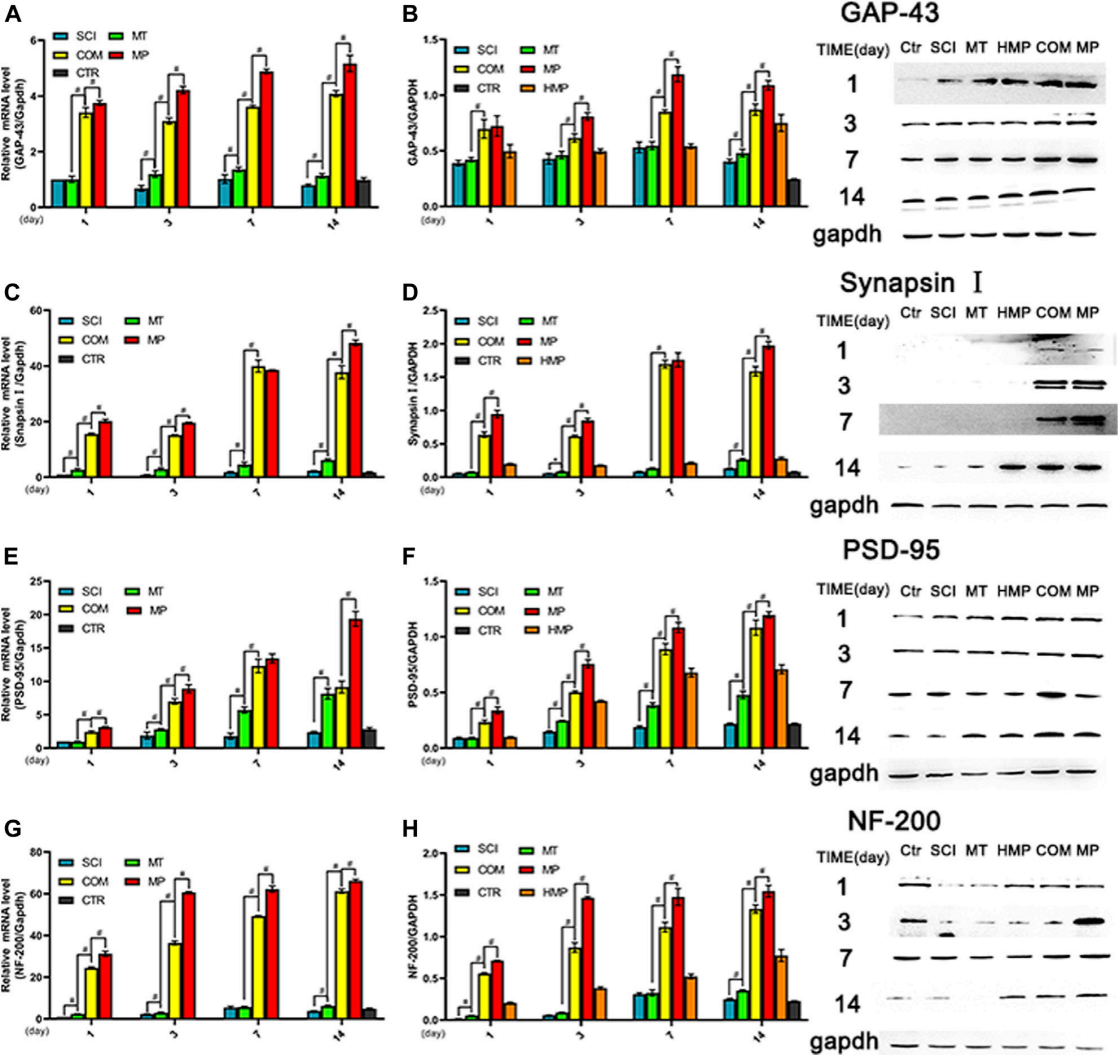
The mRNA and protein levels of key axon related proteins in rats with SCI. **(A, B)**. The relative expression levels of GAP-43 mRNA and protein (*n* = 3); **(C, D)**. The relative expression levels of synapsin I mRNA and protein (*n* = 3); **(E, F)**. The relative expression levels of PSD-95 mRNA and protein (*n* = 3). G, H. The relative expression levels of NF-200 mRNA and protein (*n* = 3), on the 1st, 3rd, 7th, and 14th days post-injury. *p < 0.05, #*p* < 0.01. GAPDH was used as the control. Quantification of these results was performed by scanning densitometry. Error bars represent the standard deviation. CTR group result is average of the 4 sampling time points.

The expression of GAP-43, NF-200, Synapsin I, and PSD-95 proteins are closely related to the recovery and plasticity of axons. The expression levels of GAP-43, synapsin I, PSD-95, and NF-200 in the COM group were significantly higher than those in the MT group at all time points but lower than those observed in the MP group (*p* < 0.01). The mRNA level of these nerve proteins in the COM group was significantly lower than those in the MP group (*p* < 0.01; Figure 4). Compared with the MT group, rats in the COM group exhibited improved axonal recovery and plasticity, but the combination’s efficacy was still lower than the standard dose of MP.

### MT Synergizes With MP to Alleviate SCI

We then analyzed the influence of various factors (MT, HMP, or time) alone or in combination on the protein expression of GAP-43, NF-200, PSD-95, and synapsin I or activation of the PI3K-AKT1 signaling pathway. These key proteins in the axon and the PI3K-AKT1 pathway were sensitive to both drugs. Both MT and HMP showed a therapeutic effect, as shown by changes in protein expression. The analysis of the main effects and interactions of the factorial design confirmed the changes in protein levels (Tables 2, 3). A synergistic effect between MT, HMP, and time was observed on axon recovery and key proteins in the PI3K-AKT1 pathway (*p* < 0.01; Tables 2, 3).

**TABLE 2:** Main effects and interaction results of ANOVA of rat neurocritical proteins.

Factor	GAP-43		Synapsin I		PSD-95		NF-200	
	*F*	*P*	*F*	*P*	*F*	*P*	*F*	*P*
MT	107.16	<0.0001	5,419.96	<0.0001	583.34	<0.0001	1,103.36	<0.0001
MP	358.66	<0.0001	7,311.50	<0.0001	2441.36	<0.0001	4402.60	<0.0001
Time	41.16	<0.0001	738.79	<0.0001	988.73	<0.0001	678.56	<0.0001
CA	46.71	<0.0001	4213.06	<0.0001	19.31	<0.0001	799.73	<0.0001
CA + Time	6.36	= 0.001	394.03	<0.0001	7.18	= 0.0004	13.67	<0.0001

**TABLE 3:** ANOVA of the PI3K-AKT1 signaling pathway in rats: main effect and interaction results.

Factor	PI3K p85		PTEN		PDK1		AKT		NF-κB	
	*F*	*P*	*F*	*P*	*F*	*P*	*F*	*P*	*F*	*P*
MT	1,263.51	<0.0001	274.29	<0.0001	2220.28	<0.0001	355.57	<0.0001	989.45	<0.0001
MP	2214.25	<0.0001	421.76	<0.0001	3996.97	<0.0001	787.96	<0.0001	1754.80	<0.0001
Time	483.57	<0.0001	248.44	<0.0001	293.81	<0.0001	134.18	<0.0001	863.72	<0.0001
CA	403.22	<0.0001	43.26	<0.0001	1.94	= 0.1697	1.65	= 0.2052	490.75	<0.0001
CA + Time	23.98	<0.0001	9.10	<0.0001	0.76	= 0.5231	40.11	<0.0001	215.04	<0.0001

Among the different effects of MT and HMP, MT could promote HMP and alter the protein concentration of key axonal proteins and the PI3K-AKT1 signaling pathway (*p* < 0.01; Tables 4, 5). In the separate effect analysis investigating the COM group and time, we found that increased treatment time can help maintain the concentration of key axonal proteins and the concentration of proteins in the PI3K-AKT1 signaling pathway in the COM group at various time points (*p* < 0.01; Tables 6, 7).

**TABLE 4:** Simple effects results of ANOVA of neurocritical proteins in rats.

Time (day)	Dosage (mg/kg)		GAP-43		Synapsin I		PSD-95		NF-200	
	MT	MP	*F*	*P*	*F*	*P*	*F*	*P*	*F*	*P*
1	0 or 15	15	42.31	<0.0001	548.71	<0.0001	45.88	<0.0001	237.70	<0.0001
3	0 or 15	15	15.04	= 0.0003	533.88	<0.0001	16.04	= 0.0002	456.22	<0.0001
7	0 or 15	15	100.98	<0.0001	6289.74	<0.0001	114.45	<0.0001	667.46	<0.0001
14	0 or 15	15	15.00	= 0.0003	4909.89	<0.0001	356.97	<0.0001	593.25	<0.0001
1	15	0 or 15	79.79	<0.0001	893.22	<0.0001	51.93	<0.0001	484.49	<0.0001
3	15	0 or 15	24.90	<0.0001	802.43	<0.0001	166.85	<0.0001	1,157.91	<0.0001
7	15	0 or 15	97.92	<0.0001	7,002.12	<0.0001	646.90	<0.0001	1,231.48	<0.0001
14	15	0 or 15	159.51	<0.0001	5,016.43	<0.0001	932.28	<0.0001	1823.08	<0.0001

**TABLE 5:** Simple effects results of variance analysis of the PI3K-AKT1 signaling pathway in rats.

Time (day)	Dosage (mg/kg)		PI3K p85		PTEN		PDK1		AKT		NF-κB	
	MT	MP	*F*	*P*	*F*	*P*	*F*	*P*	*F*	*P*	*F*	*P*
1	0 or 15	15	172.30	<0.0001	42.46	<0.0001	500.68	<0.0001	25.61	<0.0001	1.48	= 0.2287
3	0 or 15	15	140.20	<0.0001	46.47	<0.0001	89.11	<0.0001	0.50	= 0.4813	996.04	<0.0001
7	0 or 15	15	507.51	<0.0001	59.25	<0.0001	171.96	<0.0001	20.07	<0.0001	1,275.70	<0.0001
14	0 or 15	15	971.73	<0.0001	136.70	<0.0001	560.73	<0.0001	213.15	<0.0001	53.54	<0.0001
1	15	0 or 15	267.06	<0.0001	8.67	= 0.005	678.77	<0.0001	82.23	<0.0001	0.23	= 0.6311
3	15	0 or 15	252.25	<0.0001	88.24	<0.0001	310.66	<0.0001	6.27	= 0.0158	1,235.33	<0.0001
7	15	0 or 15	727.88	<0.0001	150.10	<0.0001	247.72	<0.0001	86.04	<0.0001	1885.50	<0.0001
14	15	0 or 15	1,277.44	<0.0001	189.20	<0.0001	1,021.60	<0.0001	290.17	<0.0001	132.66	<0.0001

**TABLE 6:** Simple effects of prolonged treatment time on ANOVA of neurocritical proteins.

Time factor	GAP-43		Synapsin I		PSD-95		NF-200	
	*F*	*P*	*F*	*P*	*F*	*P*	*F*	*P*
Day 1–Day 3	6.95	= 0.0112	1.53	= 0.2213	183.78	<0.0001	185.14	<0.0001
Day 3–Day 7	58.43	<0.0001	3363.53	<0.0001	381.70	<0.0001	112.84	<0.0001
Day 7–Day 14	0.34	= 0.5611	34.03	<0.0001	94.27	<0.0001	90.03	<0.0001

**TABLE 7:** Simple effects of prolonged treatment time on PI3K-AKT1 signaling pathway ANOVA.

Time factor	PI3K p85		PTEN		PDK1		AKT		NF-κB	
	*F*	*P*	*F*	*P*	*F*	*P*	*F*	*P*	*F*	*P*
Day 1–Day 3	39.92	<0.0001	25.21	<0.0001	0.61	= 0.4384	34.92	<0.0001	1,205.14	<0.0001
Day 3–Day 7	213.06	<0.0001	0.08	= 0.7727	40.29	<0.0001	0	1	16.81	= 0.0002
Day 7–Day 14	171.97	<0.0001	181.13	<0.0001	512.09	<0.0001	78.27	<0.0001	15.23	= 0.0003

## Discussion

The spinal cord connects the brain to the limbs. Injury of this fragile structure limits the information sent and received by the injured site, ultimately leading to risk of paralysis below the site of injury (Sofroniew, 2018). Cells within the injured CNS often enter a terminal differentiation state and cannot regenerate (Rouanet et al., 2017). Therefore, inhibiting apoptosis of cells in the CNS is necessary to reduce this loss of function.

Although researchers have highlighted that MP is an effective treatment to reduce nerve fiber injury, lipid peroxidation, there are still many doubts about the application of MP in the treatment of acute SCI (Silva et al., 2014; Evaniew et al., 2017; Sámano and Nistri, 2019). Recently, the Congress of Neurological Surgeons updated the ‘Systematic Review and Evidence-Based Guidelines on the Evaluation and Treatment of Patients with Thoracolumbar Spine Trauma: Pharmacological Treatment,’ suggesting that caution should be taken when using high doses of MP (Arnold et al., 2019, Fehlings et al., 2014). Therefore, this study explored whether co-administration of the melatonin could reduce the necessary dosage of MP to a sustainable concentration. Here, we aimed to clarify if co-administration of MT and a dose half that of the standard concentration of MP could elicit effects comparable to the standard dosage of MP and protect against SCI.

After SCI, Nissl bodies degrade and release cytoplasmic content, promoting cell apoptosis and resulting in secondary injury, which is a common cause of neuronal necrosis (Fan et al., 2018). Treatment with MT could stabilize Nissl bodies from day 14 after treatment. A combination of MT and low dose MP was significantly more effective at stabilizing Nissl bodies than MT alone and was comparable to the effects achieved by full-dose MP by day 7. Similarly, the combination of MT and HMP showed a synergistic effect in the prevention of apoptosis. The MP concentration in this combination did not reach the dosage used in shock therapy. Moreover, co-treatment with MT and HMP dramatically decreased the dosage of MP required to generate a therapeutic effect comparable to treatment with high-dose MP. The combination was also as effective as high-dose MP at inhibiting apoptosis.

During apoptosis, AKT1 phosphorylates and activates IKK α, degrading IκB and releasing NF-κB (Risso et al., 2015). Notably, PTEN is considered a key factor affecting CNS recovery. NF-κB inhibits PTEN generation and the release of inflammatory factors from neuronal cell body disruption (Qiu et al., 2016; Chen et al., 2017; Janku et al., 2018). Within 24 h of SCI, cell edema does not induce necrosis, limiting the release of cytotoxin cellular contents. During this time, the protein concentration of NF -κB in the MT group can inhibit PTEN. After 24 h, the protein levels of PI3K p85, PTEN, and p-PDK1 were different between groups, suggesting that the drug combination and full-dose MP exerted similar effects on AKT1 activation. This, in turn, stimulated NF-κB to degrade multiple cell endotoxins and promote cell survival. The analysis of p-NF-κB p65 showed that MT and MP functioned synergistically to activate the PI3K pathway and promote cell survival in the acute stage of SCI.

Growth-associated proteins (GAP) play essential roles in the growth and development of neurons, injury repair, axon regeneration, and synaptic remodeling (Holahan, 2017). Synapsin I and postsynaptic density (PSD)-95 are crucial for synaptic signaling function (Rocchi et al., 2019, Sheng, 2001). Neurofilament (NF)-200 is a specific marker of neurofilaments that plays an important role in maintaining NF morphology (Jackson et al., 2018). GAP-43, PSD-95, NF-200, Synapsin I. The expression levels of these four proteins indirectly reflect the state of repair after axon injury. Overall, MT and MP combination displayed an adequate therapeutic effect towards the plasticity and the repair of synapses, although it was slightly less efficacious than full-dose MP. This is consistent with the recent studies on MP that highlighted that MP mainly targets the axon.

**FIGURE 5:**
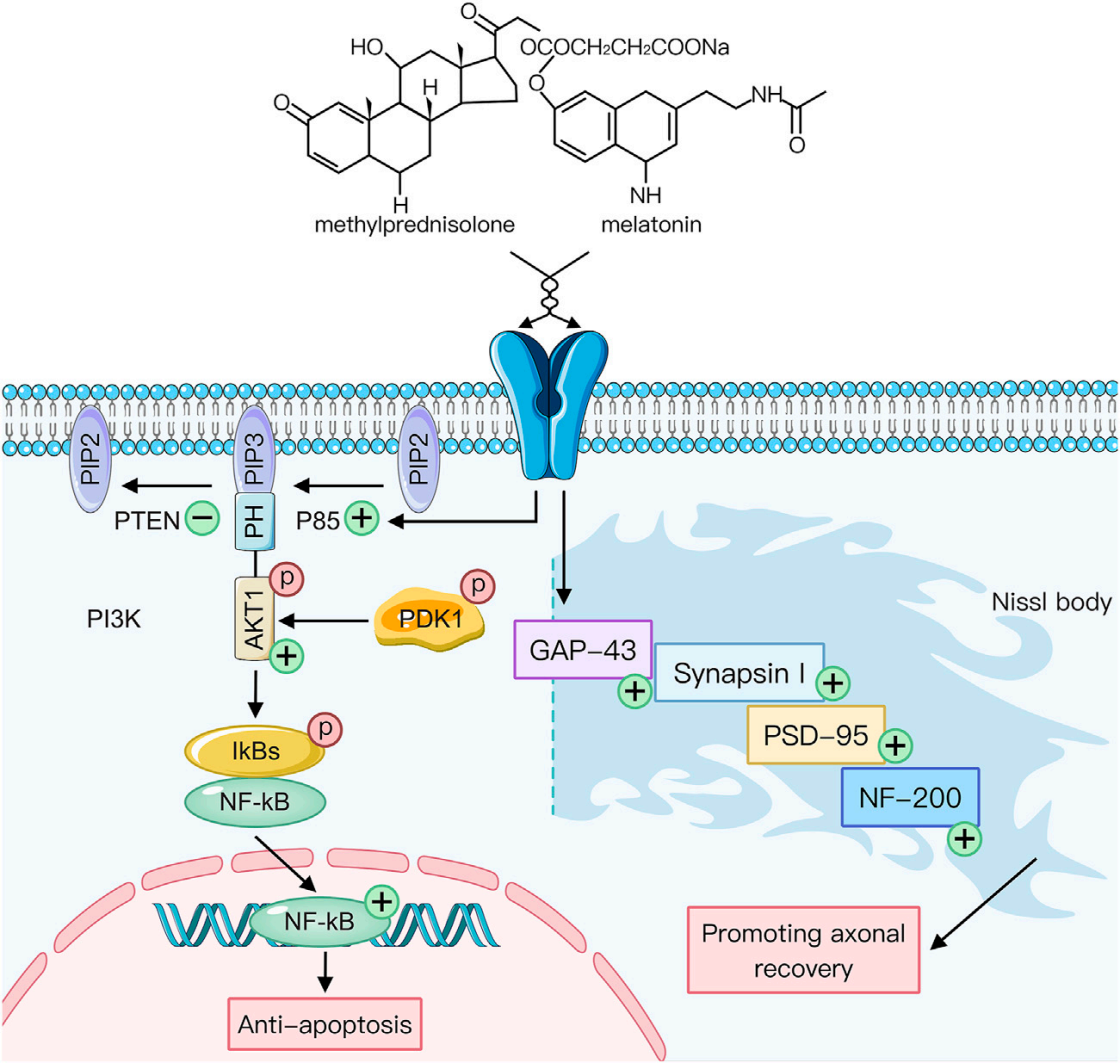
The potential mechanism underlying the synergistic effect of melatonin and methylprednisolone in the stimulation of key axon proteins and inhibition of neuronal apoptosis. Expression. Arrows: the sequential direction, +: increase; −: decrease.

At the same time, we found that MT showed some anti-apoptotic effects on neurons after spinal cord injury, but its efficacy was rather low. Simutaneously, the use of MP is questionable (Bi et al., 2021).In this study, the combination of MT with HMP significantly reduced apoptosis and showed a therapeutic effect on axons (Figure 5). MT could also activate p-AKT1 within 24 h after injury. These synergistic effects resulted in NF-κB activation 3 days after treatment. Western blot analysis showed that the therapeutic effect in the HMP group was significantly poorer compared to the MP group, but the COM group showed similar protein levels compared to the MP group. Factorial design is an effective method to observe the interaction among various factors and unveil the complex effects and relationships associated with combination therapies (Pandis et al., 2014). After calculation with a factorially designed statistical model, we found that MT had a catalytic effect on MP, enhanced MP’s anti-apoptotic effect, reduced the necessary dose of MP, and solved the issues surrounding MP’s weak effect on cell bodies. At the same time, the increase in administration time was also significant. In short, the synergistic effect of MT and HMP in the treatment of spinal cord injury was shown by a reduction in neuronal apoptosis and adverse drug reactions caused by MP.

## Data Availability

The original contributions presented in the study are included in the article/Supplementary Material, further inquiries can be directed to the corresponding author.
